# Impact of High-Pressure Processed Onion on Colonic Metabolism Using a Dynamic Gastrointestinal Digestion Simulator

**DOI:** 10.3390/metabo11050262

**Published:** 2021-04-22

**Authors:** Irene Fernández-Jalao, Claudia Balderas, María V. Calvo, Javier Fontecha, Concepción Sánchez-Moreno, Begoña De Ancos

**Affiliations:** 1 Department of Characterization, Quality and Safety, Institute of Food Science, Technology and Nutrition (ICTAN-CSIC), 28040 Madrid, Spain; irene.fernandez@ictan.csic.es (I.F.-J.); claudia.balderas03@gmail.com (C.B.); csanchezm@ictan.csic.es (C.S.-M.); 2 Department of Bioactivity and Food Analysis, Institute of Food Science Research (CIAL, CSIC-UAM), 28049 Madrid, Spain; mv.calvo@csic.es (M.V.C.); j.fontecha@csic.es (J.F.)

**Keywords:** gastrointestinal digestion-colon fermentation model, high-pressure processing, onion, quercetin glycosides metabolites, gut microbiota, short-chain fatty acids

## Abstract

Onions are the main dietary source of flavonols that have been associated with important health-promoting properties. Onion treated by high-pressure processing (HPP-treated onion) was subjected to a dynamic gastrointestinal digestion and colon fermentation simulator (DGID-CF) to study the effect on the gut microbiota metabolism in the three colon regions (ascending—AC, transverse—TC, and descending—DC) by means of chronic feeding with 27 g/day for 14 days. HPP-treated onion presented a high content of the flavonols quercetin-3,4’-diglucoside and quercetin-4’-glucoside, and a large percentage of them reached the AC without change. TC and DC progressively increased the total phenolic metabolites 2.5 times respective to day 2, mainly 3-hydroxyphenylacetic, 4-hydroxyphenylacetic, 3-(4-hydroxyphenyl)-propionic, and 3,4-dihydroxyphenylpropionic acids. In addition, the chronic feeding increased the beneficial colon bacteria *Bifidobacterium* spp. and *Lactobacillus* spp. and the production of total SCFAs (acetic, propionic, and butyric acids) 9 times (AC), 2.2 times (TC), and 4.4 times (DC) respective to day 1. A multivariate analysis (principal component analysis, PCA) showed a clear separation between the three colon regions based on their phenolic composition (precursors and metabolites). These results showed that HPP-treated onion modulated the human gut microbiota’s metabolism and the DGID-CF is a good system to study these changes.

## 1. Introduction

Onions (*Allium cepa* L.) are the second main crop grown worldwide after tomatoes with a current production estimated at 96.77 million tons [1] and are considered a primary ingredient of major worldwide diets [2]. Onions are also an essential source of nutrients and bioactive compounds (such as phenolic and organosulfur compounds) and dietary fibers (mainly oligosaccharides) [3]. Daily onion consumption has long been associated with health-promoting properties including antimicrobial, antibiotic, antioxidant, analgesic, antiplatelet, antithrombotic, anti-inflammatory, antidiabetic, anticarcinogenic, hypolipidemic, antihypertensive, hepatoprotective, and immunoprotective effects [2,3]. The health-protecting effects of onion consumption have been extensively related with their phenolic composition, mainly flavonols, a subgroup of flavonoids [4,5]. Onion is one of the main dietary sources of the flavonol quercetin (Q) along with apples. In onions, quercetin is linked to glucose in the form of monoglucosides such as Q-4′-O-glucoside, diglucosides such as Q-3,4′-di-O-glucoside, and triglucosides such as Q-3,7′,4′-tri-O-glucoside [6]. The beneficial properties of quercetin are closely related to its chemical structure, which gives it excellent antioxidant power to act against reactive oxygen species and free radicals and inhibit pro-oxidant enzymes (lipoxygenase, xanthine oxidase, NADPH oxidase) [7]. Moreover, quercetin has shown cardioprotective effects related to its anti-inflammatory activity, inhibition of LDL oxidation, reduction of adhesion molecules, platelet antiaggregating, and vasodilator effects [8,9]. 

However, to exert the biological functions of flavonoids, they must be first released from the food matrix during gastrointestinal digestion and become available (bioaccessible) for further intestinal absorption, and later, reach the target organ or systemic circulation (bioavailable). Bioaccessibility and the absorption of flavonoids are determined by different factors such as chemical structure, degree of polymerization or glycosylation, solubility and conjugation with other phenolics, the food matrix composition, possible interactions with other food components present in the diet (carbohydrates, lipids, proteins, fiber, etc.) [10], and food processing technologies such as high-pressure processing (HPP) [11]. HPP has been proposed as an alternative to thermal processing technologies to produce high-quality, safe, and fresh-like plant-derived foods. HPP promotes microbiological and enzymatic inactivation in vegetables and fruits, and thus this technology preserves the compounds responsible for their sensory and nutritional quality and health-promoting characteristics [11]. Moreover, certain HPP treatments induce the breakage of cellular structures allowing the release of phenolic compounds into the extracellular medium, thus increasing their bioaccessibility [11]. 

The recovery rates of flavonoids in plasma and urine in most absorption studies are usually much less than 50% of the initial content in the food, showing their low bioaccessibility [12]. Accordingly, the majority of flavonoids reach the colon intact, where they undergo an extensive metabolism by the gut microbiota [13,14]. The microbial metabolism of flavonoids in the colon depends on the specific abilities of the bacteria strains to cleave certain chemical bonds [15]. Thus, flavonoid glycosides are transformed into their corresponding aglycones by the action of microbiota enzymes, α-rhamnosidases, β-glucosidases, and β-glucuronidases [16]. Then, flavonoid aglycones undergo different transformations including C-ring breakdown, α-oxidation, β-oxidation, dehydrogenation, demethylation, and dehydroxylation [17]. These transformations generate a wide range of low molecular weight phenolic acids as metabolites that are structurally different and potentially more biologically active than their parent’s compounds. In general, the main phenolic acid metabolites are hydroxylated forms of phenylpropionic, phenylacetic, and benzoic acids, among others. In fact, the health benefits related to the intake of flavonoids could be mainly attributed to their bioactive metabolites [17,18]. Thus, quercetin colonic metabolites such as 3,4-dihydroxyphenylacetic acid, 3,4-dihydroxyphenylpropionic acid, and homovanillic acid (3-methoxy-4-hydroxyphenyl acetic) have shown an antioxidant activity even higher than other antioxidant compounds such as α-tocopherol [19]. Furthermore, 3,4-dihydroxyphenylpropionic acid (dihydrocaffeic acid), 3-(4-hydroxy-3-methoxyphenyl)-propionic acid (hydroferulic acid), and 3,4-dihydroxyphenylacetic acid have been investigated by their important in vitro and in vivo anti-inflammatory effects [20]. Moreover, 3,4-dihydroxybenzoic acid (protocatechuic acid) has demonstrated in vitro and in vivo properties against carcinogenesis and cardiovascular and neurodegenerative diseases [21,22].

Phenolic compounds and dietary fiber usually enter unchanged and simultaneously into the colon, being both the main substrates that lead to the formation of phenolic acids and short chain fatty acids (SCFAs), respectively, as a consequence of the catabolism of the intestinal microbiota [23,24]. The main SCFAs are acetic, butyric, and propionic acids [25]. SCFAs play an essential role in the novo synthesis of lipids and glucose in the cells, in the production of gut hormones, in the brain to regulate food intake, in the protection against enteric pathogen colonization and infection, and in the prevention and treatment metabolic diseases, among other functions [26]. Butyric and propionic acids protect against diet-induced obesity, regulate gut hormones, and reduce food intake [27]. In addition, butyric acid has been evaluated by its capacity to prevent malignant cells’ formation and induce apoptosis in colonic cancer cells [28], and acetic acid contributes to lipogenesis in several tissues such as liver [26]. However, several studies reported that acetic acid promoted obesity via hyperphagia and insulin secretion in rodents [29], and propionic acid impaired insulin action via glucagon secretion [30].

The health-promoting effect of phenolic compounds and their microbial metabolites could be reflected in the modulation of beneficial bacteria and their metabolic activity through the increase in SCFA production. The non-absorbed phenolic compounds and their metabolites may contribute to intestinal health by modulating microbiota composition through selective prebiotic effects and antimicrobial activities against pathogenic bacteria [13]. The relationship between the phenolic metabolites and SCFA production could be associated with a possible modulation effect on carbohydrate-fermented bacteria growth [17]. However, the mechanisms by which phenolic compounds modulate the gut microbiota are still unclear. Some studies have revealed that metabolites derived from phenolic compounds (purees or as food constituents) stimulated the proliferation of beneficial bacteria such as *Bifidobacterium* spp. and *Lactobacillus* spp. and decreased that of *Clostridium* spp., which are considered harmful [24,31]. A balanced microbiota composition is related to maintaining good health; meanwhile, the imbalance, also called dysbiosis, is related with some health disorders such as intestinal bowel disease, irritable bowel syndrome, colorectal cancer, obesity, diabetes, metabolic syndrome, and cardiovascular disease, among others [32]. The intake of phenolic-rich sources has been proposed to prevent or even reverse the dysbiosis associated with different pathologies [33].

Although in vivo human or animal intervention trials are physiologically most relevant to study both flavonoid metabolism and microbial modulation, in vitro tools have been designed to simulate intestinal conditions because in vivo studies often imply a long time and a high cost [34]. In vitro models range from simple fermenters to highly complex models such as dynamic multistage models designed to better reproduce both human gastrointestinal digestion and the colonic fermentation process [35]. Dynamic multistage fermentation models are designed to simulate the biochemical (pH changes, temperature, anaerobic conditions) and mechanical conditions (peristaltic movements, retention time, digested transit) of the whole digestion and fermentation process, as well as the metabolic conditions (microbial growth rate and substrate availability) in the different colon regions (ascending, transverse, and descending colon) [36]. These models have been applied to study the effect of foods rich in phenolic compounds on the gut microbiota metabolism [37,38,39].

Previous in vivo assays with Wistar rats have demonstrated that HPP-treated onion (400 MPa/25 °C/5 min) improved antioxidant defense mechanisms, reduced the level of hepatotoxicity, and improved the inflammatory response associated with atherogenesis in hypercholesterolemic rats. Furthermore, supplementation with the HPP-treated onion induced an increase in *Lactobacillus* spp. and *Bifidobacterium* spp., while the pathogens *Clostridium* spp. decreased in comparison with rats fed with normal and high-cholesterol diets [40,41].

The aim of the current study was to evaluate the impact of chronic feeding for 14 days with HPP-treated onion (400 MPa /25 °C/5 min) on colonic metabolism using a dynamic in vitro gastrointestinal digestion and colonic fermentation (DGID-CF) simulator inoculated with the human feces of healthy volunteers. The gut metabolic activity was carried out by analyzing the flavonol compounds and their metabolites, the formation of SCFAs, and the changes in the microbial population in the different colon regions (ascending, transverse, and descending colon).

## 2. Results and Discussion

### 2.1. Physicochemical and Chemical Characterization and Phenolic Compounds of Undigested HPP-Treated Onion Powder

Table 1 shows the organic acids, sugars, and fiber content of the undigested HPP-treated onion powder. These data agree with other results previously published for onions by other authors [42,43,44]. Furthermore, Table 1 shows the phenolic compounds content of the undigested HPP-treated onion that was separated, identified, and quantified by high performance liquid chromatography-mass spectrometry system with a diode array detector (DAD) and coupled to an accurate-mass quadrupole time-of-flight (Agilent 6520I Accurate-Mass Q-TOF LC/MS) equipped with a jet stream dual electrospray ionization (ESI) interface (HPLC-DAD-ESI-QTOF-MS/MS) (Agilent Technologies, Santa Clara, CA, USA) according to the procedure described by Fernandez-Jalao et al. [45]. The phenolic compound composition was similar to those previously described for onions [46]. The main phenolic compounds of undigested HPP-treated onion were flavonols, and the major ones were quercetin-3,4′-di-*O*-glucoside (Q-3,4′-diglucoside) (75.3%), quercetin-4′-*O*-glucoside (Q-4′-glucoside) (15%), and isorhamnetin-4′-*O*-glucoside (Iso-4′-glucoside) (4.8%) (Table 1). The remaining flavonols represented 5% of total flavonol content, which was 6765.16 ± 362.27 µg/g dw. In addition, protocatechuic acid, salicylic acid, and gallic acid were identified in the HPP-treated onion at very low concentration (≤2 µg/g dw) [44,47].

### 2.2. Phenolic Compounds and Metabolites in the Colon Fermentation Products of HPP-Treated Onion Powder. Metabolomic Analysis by a Targeted Approach

#### 2.2.1. Evolution of Flavonol Glycosides during Colonic Fermentation

**Figure 1:**
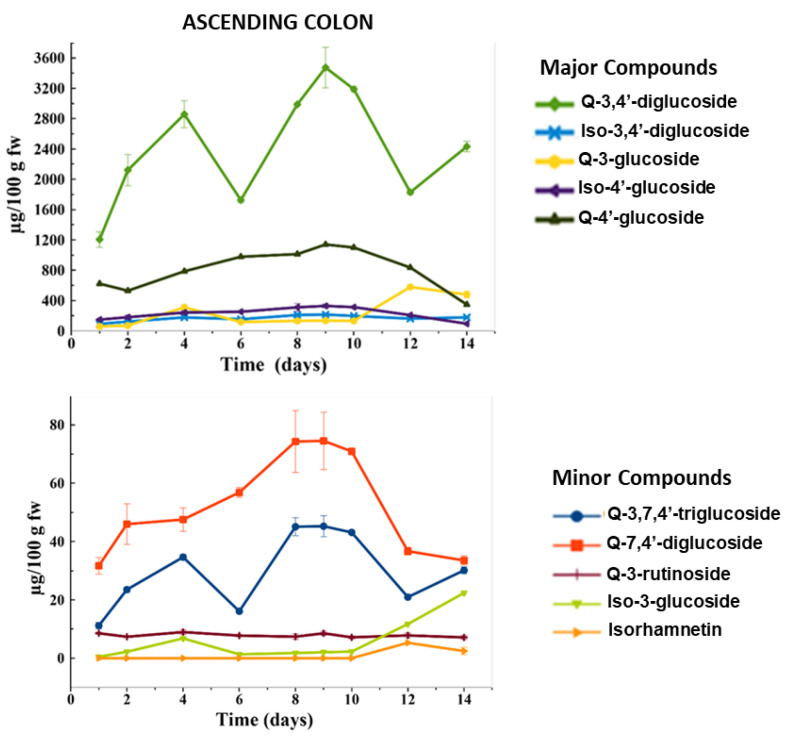
Concentration (µg/100 g fw of slurry) of flavonols in the ascending colon (AC) during the colonic fermentation of HPP-treated onion. Data are the mean value ± standard deviation of the three different determinations (*n* = 3).

Some of the flavonol glycosides were separated, identified and quantified in the undigested HPP-treated onion powder (Table 1), as well as in the gut fermentation samples of the ascending colon (AC) (Table 2) (Figure 1) by HPLC-DAD-ESI-QTOF-MS/MS. At the initial stage of the fermentation (day 1), the major flavonols found in the fresh AC slurry were Q-3,4′-diglucoside (1205.88 ±102 µg/100 g fw of slurry) and Q-4′-glucoside (622.46 ± 1.26 µg/100 g fw of slurry), which were also the major flavonols found in the undigested HPP-treated onion (Table 1). Moreover, Iso-4′-glucoside (148.65 ± 4.86 µg/100 g fw of slurry), Iso-3,4′-diglucoside (92.75 ± 6.65 µg /100 g fw of slurry), and Q-3-glucoside (59.06 ± 2.05 µg/100 g fw of slurry) were determined at the beginning of the study in the AC.

Other onion flavonols were found at concentrations lower than 30 µg/100 g fw of slurry (Q-3,7,4′-triglucoside, Q-7,4′-diglucoside, Q-3-rutinoside, Iso-3-glucoside, and Isorhamnetin aglycone). During the gut fermentation study of 14 days with the HPP-treated onion, the major flavonols (Q-3,4′-diglucoside and Q-4′-glucoside) were accumulated daily in the AC slurry, reaching a maximum on day 9 (3472.87 ± 269 and 1139.42 ± 100 µg/100 g fw of slurry, respectively), following which they decreased their concentration progressively until the end of the intake period (day 14) (Figure 1). A similar trend was followed by other minor flavonols (Iso-4′-glucoside, Iso-3,4′-diglucoside and Q-7,4′-diglucoside) that also showed their maximum concentration on day 9 of the treatment period, reaching 2 times the concentration of day 1. This behavior observed for the majority of flavonol glycosides in the AC suggests that during the first 9 days of the study, the accumulation of flavonol glycosides predominated over the fermentation process. The accumulation of Q-3-glucoside and Iso-3-glucoside after 14 days of chronic feeding could be a consequence of the degradation of the major compounds Q-3,4′-diglucoside and Iso-3,4′-diglucoside, respectively, which was more intense from day 9 to the end of intake period. This behavior could also be related to the fact that some colonic bacterial species are not capable of degrading flavonols with sugar moieties at 3-O, 5-O, or 7-O position [15].

These results showed that continuous feeding with the HPP-treated onion may cause an accumulation of flavonols in the AC. This result agrees with the fact that the bioaccesibility of total flavonols in the HPP-treated onion (400 MPa/25 °C/5 min) was 17.47% (previously studied by research group) [46]. Therefore, approximately 83% of the flavonols present in the HPP-treated onion were able to reach the AC unchanged and available to be subjected to the gut microbiota metabolism [48].

The results obtained with a chronic feeding with 27 g per day of HPP-treated onion powder employing a DGID-CF simulator differed from those obtained from static models feeding with a single dose of phenolic compounds standards, which suffer a rapid biotransformation, disappearing from the medium within 0.5 h and being totally replaced by different phenolic acids that have been identified as their corresponding metabolites [24].

#### 2.2.2. Evolution of Flavonol Glycosides during Colonic Fermentation

The dynamic gastrointestinal digestion-colon fermentation (DGID-CF) system was fed every day with 27 g of HPP-treated onion powder representing, a daily intake of 150.39 µg of quercetin aglycone according to its initial content (Table 3).

After gastrointestinal digestion and taking into account quercetin bioaccessibility (10.47%) in the HPP-treated onion determined in previous studies [46], the amount of quercetin that reached the colon unaltered could be much less, about 134.64 µg per day (89.53% of 150.39 µg) (Table 3). This low amount of quercetin that fed the colon daily was not consistent with the high quercetin concentration found in the AC (Table 3), which could be most related to the deglycosylation of quercetin glycosides by gut microbiota [5]. Contrary to that observed for quercetin glycosides, quercetin aglycone was also present in the transverse colon (TC) and descending colon (DC), but in a significantly lower concentration (Table 3).

These results show that the deglycosylation of quercetin glycosides, probably catalyzed by gut microbial enzymes such as β-glucosidases, β-glucuronidases, and α-rhamnosidases [16], took place with greater intensity in the AC than in the TC and DC, resulting in the quercetin aglycone being released. The very low concentration of quercetin aglycone found in the TC and DC could indicate that in these two regions of the colon, the degradation of quercetin aglycone by the gut microbiota predominates. In general, a quick deglycosylation of quercetin glycosides has been described in different plant-derived foods submitted to fecal fermentation using static models. Thus, quercetin glycosides from green pepper, mainly quercetin-3-rhamnoside, were quickly metabolized in the first minutes of fecal fermentation while accumulating quercetin in high concentration, reaching the maximum concentration after 5 h [49]. Moreover, quercetin glycosides from apples, mainly quercetin-3-glucoside and quercetin-3-rhamnoside, were completely transformed in quercetin aglycone after 24 h of the fecal fermentation of apples [23]. These results agree with the fact that the quercetin deglycosylation may occur at different rates depending on its concentration [50], sugar moiety [16], and inter-individual variation of the microbiota profile [51]. Thus, the food matrix could modulate the interaction of the phenolic compound with the gut microbiota [17,52].

The use in the present study of a dynamic fermentation model system that was continuously fed with the HPP-treated onion allowed the presence of great amounts of quercetin aglycone during the 14 days of the study, which promotes important health-protective characteristics to the colon, mainly in the AC. Quercetin presented great antioxidant power against reactive oxygen species and free radicals, and the capacity to inhibit pro-oxidant enzymes (lipoxygenase, xanthine oxidase, NADPH oxidase) [7]. Moreover, quercetin has shown cardioprotective effects related to its anti-inflammatory activity, capacity of inhibition of LDL and oxidation and reduction of adhesion molecules, platelet antiaggregating, and vasodilator effects [8,9].

#### 2.2.3. Evolution of Flavonol Metabolites during Colonic Fermentation

In general, the gut microbiota metabolism of phenolic compounds involves numerous reactions (hydrolysis, hydrogenation, α- and β-oxidation, demethoxylation, dehydroxylation, and decarboxylation) that result in the formation of a wide range of metabolites [18]. The non-bioaccessible flavonol glycosides of the HPP-treated onion that reached the ascending colon (AC) unchanged after the gastrointestinal digestion suffered firstly a deconjugation catalyzed by fecal microbiota enzymes (ß-glucosidase, α-rhamnosidase, and ß-glucuronidase), releasing the quercetin aglycone that was then extensively degraded by the colon microbiota to produce a wide range of metabolites, mainly phenolic acids.

**Figure 2:**
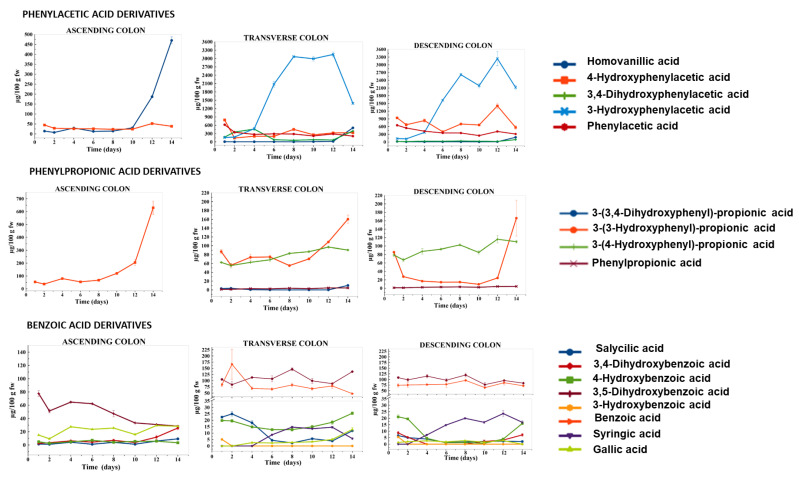
Concentration (µg/100 g fw of slurry) of phenylacetic, phenylpropionic, and benzoic acid derivatives, in the ascending colon (AC), transverse colon (TC), and descending colon (DC) during the colonic fermentation of HPP-treated onion powder. Data are the mean values ± standard deviation of three different determinations (*n* = 3).

A total of 23 phenolic metabolites were identified by HPLC-DAD-ESI-QTOF-MS/MS (Table 2) and were targeted during the different regions of the gut fermentation dynamic model feeding for 14 days with the intestinal digestion fraction of the HPP-treated onion. In decreasing order of concentration by families, derivatives of phenylacetic acids, phenylpropionic acids, benzoic acids, and others were identified (Table 2). Some of these microbial metabolites appeared in the ascending colon (AC) even if they were found in higher concentration in the transverse colon (TC) and descending colon (DC), along with the disappearance of parent phenolic compounds. The evolution of phenolic compound metabolites, described by families, during the feeding intake period with 27 g of HPP-treated onion (once a day for 14 days) in the three colon regions, ascending colon (AC), transverse colon (TC), and descending colon (DC), is shown in Figure 2 and described below.

**Phenylacetic acid derivatives**. This group belongs to the major metabolite found in the present study: 3-hydroxyphenylacetic acid. Aside from this metabolite, the concentration of 3,4-dihydroxyphenylacetic acid, 4-hydroxyphenylacetic acid, homovanillic acid (3-methoxy-4-hydroxyphenylacetic acid), 2-hydroxyphenylacetic acid (tentatively identified), and phenylacetic acid in the three regions (AC, TC, and DC) of the gut fermentation dynamic model fed with the HPP-treated onion for 14 days were shown in Figure 2.

3,4-Dihydroxyphenylacetic acid was not detected in the AC, but in TC and DC, it was found in increasing concentration from day 1 (176.08 and 18.75 µg/100 g fw of slurry, respectively) to a peak content on day 4 in both regions (462.66 and 136.47 µg/100 g fw of slurry, respectively), remaining stable afterwards (Figure 2).

In addition, 3-hydroxyphenylacetic acid, formed by dehydroxylation from 3,4-dihydroxyphenylacetic acid was not found in the AC, but the concentration of this metabolite increased progressively from day 1 to day 8–10, reaching a peak concentration of approximately 3000 µg/100 g fw in TC and 2700 µg/100 g fw in the DC (Figure 2). This result agrees with previous studies that showed 3-hydroxyphenylacetic acid as the major metabolite of quercetin submitted to an in vitro static fermentation process [24].

However, 4-hydroxyphenylacetic acid, also formed from 3,4-dihydroxyphenylacetic acid, was found in AC, TC, and DC. The AC presented a low concentration (average of 25 µg/100 g fw) of slurry during most of the fermentation process (Figure 2). The concentration of this metabolite significantly increased at day 1 in the TC (790.51 ± 16.81 µg/100 g fw of slurry) and DC (939.45 ± 8.54 µg/100 g fw of slurry), and then descended in successive days until day 12, reaching peak concentration in the two colon regions (939.45 ± 8.54 and 1406.1 ± 4.97 µg/100 g fw in TC and DC, respectively) (Figure 2).

Another metabolite tentatively identified as 2-hydroxyphenylacetic, which could be formed from 3- and 4-hydroxyphenylacetic acids, showed a peak concentration at day 1 in the AC (84.84 ± 6.96 µg/100 g fw), TC (776.34 ± 21.96 µg/100 g fw), and DC (848.33,33 ± 29.99 µg/100 g fw). A decrease of 48% at day 2 in comparison to day 1 was observed in the three regions of the colon reaching an average concentration of 48, 387, and 420 µg/100 g fw in the AC, TC and DC, respectively, from day 3 to day 14 of the intake period (Figure 2).

Phenylacetic acid was only detected in the TC and DC, showing a decreasing trend from day 1 to day 14 (Figure 2). This behavior indicated that this metabolite could be present in the initial fecal samples before the chronic feeding with HPP-treated onion as a result of the metabolism of the gut microbiota. Similar results were found by other authors that studied a chronic feeding of a gut fermentation model system with grape pomace [53].

3-Methoxy-4-hydroxyphenylacetic acid (homovanillic acid) was found in the three colon regions but in low concentration (<30 µg/100 g fw) during 11 days of the intake period. From day 11 to 14, a significant increase of 15, 50, and 24 times the concentration in the AC, TC, and DC, respectively, was observed.

Phenylacetic acid derivatives have been found in the three regions of the colon, specifically di- and mono-hydroxylated phenylacetic acids, which have been described as the main gut microbial metabolites from quercetin and quercetin glycosides [5,16]. The degradation pathway of quercetin starts with the formation of dihydroquercetin, which is further degraded into 3-(3,4-dihydroxyphenyl)-propionic (both identified in the present study), followed by a C-ring fission. Successive degradations form the 3,4-dihydroxyphenylacetic acid, which may be dehydroxylated to monohydroxyphenylacetic acids and phenylacetic acids, or methylated to form homovanillic acid [5,17]. Phenylacetic acids derivatives have also been identified as metabolites of other flavonoids (i.e., flavan-3-ols), but not as main metabolites [24,54].

**Phenylpropionic acid derivatives**. The major metabolite of this group was 3-(3-hydroxyphenyl)-propionic acid, which was in the three regions of the colon in a stable concentration in AC (~58 µg/100 g fw of slurry), TC (~76 µg/100 g fw of slurry), and DC (~18 µg/100 g fw of slurry) during the first 8 days of the study (Figure 2). The concentration of this metabolite significantly increased from day 10 to day 14, reaching a maximum in the AC (629.28 ± 51.95 µg/100 g fw of slurry), TC (160.22 ± 9.33 µg/100 g fw of slurry), and DC (166.07 ± 42.6 µg/100 g fw of slurry) (Figure 2).

Moreover, 3-(4-hydroxyphenyl)-propionic acid was found in the TC at a similar concentration to 3-(3-hydroxyphenyl)-propionic acid during most days of chronic feeding with HPP-treated onion (Figure 2). However, 3-(4-hydroxyphenyl)-propionic acid was the major compound in the DC and its concentration increased from day 1 (~78 µg/100 g fw of slury) to day 14 (~100 µg/100 g fw of slurry) (Figure 2). These two metabolites were formed from 3-(3,4-dihydroxyphenyl)-propionic acid that was detected in the TC at the end of the intake period but in very low concentration (10.21 µg/100 g fw of slurry) (Figure 2). This compound could be dehydroxylated and transformed to 3-(4-hydroxyphenyl)-propionic acid and 3-(3-hydroxyphenyl)-propionic acid, and this last could have suffered a subsequent transformation to produce 3-hydroxyphenylacetic acid, the most abundant metabolite found in the present study, mainly observed in the TC and DC (Figure 2).

Phenylpropionic acid derivatives are common metabolite products of the degradation pathways of different phenolic compound families in addition to flavonoids. Thus, after the deconjugation of flavonoids and subsequent aglycones release and de-esterification of phenolic acids, latter transformations (i.e., dehydroxylation, reduction, and C-ring fission) allow the formation of phenylpropionic acid derivatives [17,18].

**Benzoic acid derivatives**. The benzoic acid derivatives identified could be divided into two groups: the majority, a dihydroxybenzoic acid tentatively identified as 3,5-dihydroxybenzoic acid (Table 2) and benzoic acid; and the minority, such as 3,4,5-trihydroxybenzoic acid (gallic acid), 3,4-dihydroxybenzoic acid (protocatechuic acid), 4-hydroxybenzoic acid, 2-hydroxybenzoic acid (salicylic acid), and 4-hydroxy-3,5-dimethoxybenzoic acid (syringic acid) (Figure 2).

The most abundant benzoic acid found in the AC, TC, and DC of the present study was 3,5-dihydroxybenzoic acid followed by benzoic acid but mainly in the TC and DC (Figure 2). The concentration of 3,5-dihydrozybenzoic acid decreased approximately 64% in the AC from day 1 (77.53 ± 4.43 µg/100 g fw of slurry) to day 14 (28.44 ± 41.21 µg/100 g fw of slurry), while 3,4-dihydroxybenzoic acid (protocatechuic acid) increased from day 1 (2.48 µg/100 g fw of slurry) to day 14 (25 µg/100 g fw). In the TC and DC, 3,4-dihydroxybenzoic acid was detected but in a very low concentration (<8 µg/100 g fw of slurry). 3,5-Dihydrozybenzoic acid and benzoic acid were the major metabolites of this group, with a peak level on day 8 in TC (146.56 and 82.87 µg/100 g fw of slurry, respectively) and in DC (119.74 and 95.96 µg/100 g fw of slurry), respectively.

4-Hydroxybenzoic acid was more abundant in the TC than in AC and DC and its concentration was relatively stable (average 22.5 µg/100 g fw) during the 14 days of the intake period (Figure 2). This compound showed an irregular evolution during fermentation in the three colon regions.

Gallic acid was found in the three colon regions, especially in the AC. Gallic acid is a common phenolic compound in vegetables, and its accumulation in the AC could also be related to the presence of this compound in the initial fecal product before feeding the system with HPP-treated onion, due to this acid being the end product of the gut fermentation of different phenolic families such as gallotannins.

3,4-Dimethoxybenzoic acid (syringic acid) that was detected in the TC and DC from day 4 to 14 could be formed from gallic acid by the action of gut bacteria enzymes [55].

2-Hydroxybenzoic acid (salicylic acid) was observed in the HPP-treated onion, albeit at low concentrations (1.87 µg/100 fw of slurry), but in the fermentation products, a trend of its content increasing was observed in the AC. However, in the TC and DC, salicylic acid content was stable or instead decreased towards the end of the study. Moreover, salicylic acid has been observed after the fermentation of an extract of grape and cranberry with gut microbiota [38]. However, contrary to our results, the increase of salicylic acid was observed towards the end of the incubation period (48 h) in the DC.

Benzoic acid derivatives are amongst the end-products of the metabolism of phenolic compounds due to the gut microbiota [21,54]. 3,4-Dihydroxybenzoic acid could be formed through the B-ring degradation of some phenolic compounds such as anthocyanins or quercetin [21,47]. In addition, 3,4-dihydroxyphenylacetic acid generated from the catabolic pathways of different phenolic compounds could be degraded into 3,4-dihydroxybenzoic acid (protocatechuic acid), and finally into benzoic acid [17,56].

**Other metabolites**. Two simple phenolic compounds were identified and quantified in the three colonic fermentation regions: catechol and phloroglucinol. The concentration of phloroglucinol was higher in the AC, mainly during the first 8 days of the intake period (~350 µg/100 g fw of slurry) than in the TC (average 74 µg/100 g fw of slurry) and DC (average 62 µg/100 g fw of slurry). In general, phloroglucinol resulted from an end-product from the metabolism of quercetin and other compounds such as procyanidins, chalcones, and dihydrochalcones [18,56,57]. In the case of HPP-treated onion, phloroglucinol could be one of the end metabolites of flavonols with hydroxyl groups at position 5 and 7 of the A-ring [54]. Catechol was quantified during the entire intake period in the three colon regions in both ingredients. The concentration was similar between the AC, TC, and DC in both ingredients. Catechol could be formed by the decarboxylation of protocatechuic acid (3,4-dihydroxybenzoic acid) [58].

Furthermore, dihydroquercetin was detected at the end of the intake period in the AC and TC. This compound, also called taxifolin, has been described as an initial metabolite formed from quercetin which could be further metabolized to di- and mono-hydroxyphenylpropionic acids [17].

**Figure 3:**
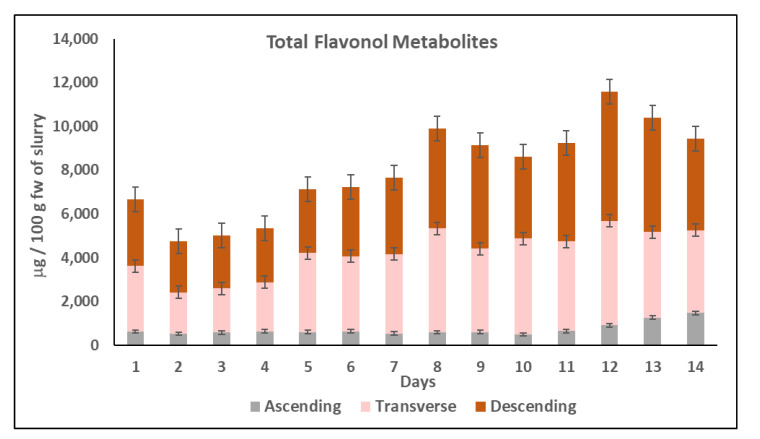
Total flavonols metabolites (µg/100 g fw of slurry) in the ascending colon (AC), transverse colon (TC), and descending colon (DC) during the colonic fermentation of HPP-treated onion. Data are the mean values ± standard deviation of three different determinations (*n* = 3).

**Total flavonol metabolites**. The evolution of total flavonol metabolite content identified in the three colon regions during chronic feeding with HPP-treated onion is shown in Figure 3. The flavonol metabolites were mainly formed in the TC and DC and their concentration was between 3 and 4 times higher than in the AC. During the 14 days of the chronic feeding study, the total metabolites content increased from day 2 to a maximum concentration at day 12 in TC (4779.34 ± 179 µg/100 g fw of slurry) and in DC (5910.61 ± 165 µg/100 g fw of slurry) which represented an increase of 2.5 times the initial concentration (Figure 3).

Different health-promoting effects of some of the flavonol metabolites found in the gut fermentation products of HPP-treated onion in the current study have been previously described. Thus, 3,4-dihydroxyphenylacetic acid has shown in vitro and in vivo antiproliferative [59], hepatoprotective [60], anti-inflammatory [61], and antioxidant properties [62]. In addition, 3-(3-hydroxyphenyl)-propionic acid has shown its potential as a vasodilator, even more than quercetin [63]. In fact, this HPP-treated onion powder (400 MPa/25 °C/5 min) supplied as a supplement in the diet of hypercholesterolemic Wistar rats for 7 weeks demonstrated antioxidant, anti-inflammatory, hepatoprotective properties and the capacity to prevent or partially modulate vascular dysfunction by reducing some of the risk indexes linked to the initial stages of atherosclerosis [40,41]. These health-promoting properties of the HPP-treated onion ingredient demonstrated by in vivo assays with rats could be related with the formation in the gut of the different metabolites described in the present study, such as 3,4-dihydroxyphenylacetic acid mainly found in the TC and DC, although it was quickly metabolized to 3-hydroxyphenylacetic resulting in the major metabolite (Figure 2). In general, the different phenolic metabolites profile in the three colon regions (AC, TC, DC) could be attributable to different microbiota composition among them [64].

#### 2.2.4. Multivariate Data Analysis of the HPLC-DAD-ESI-QTOF-MS/MS Data

**Figure 4:**
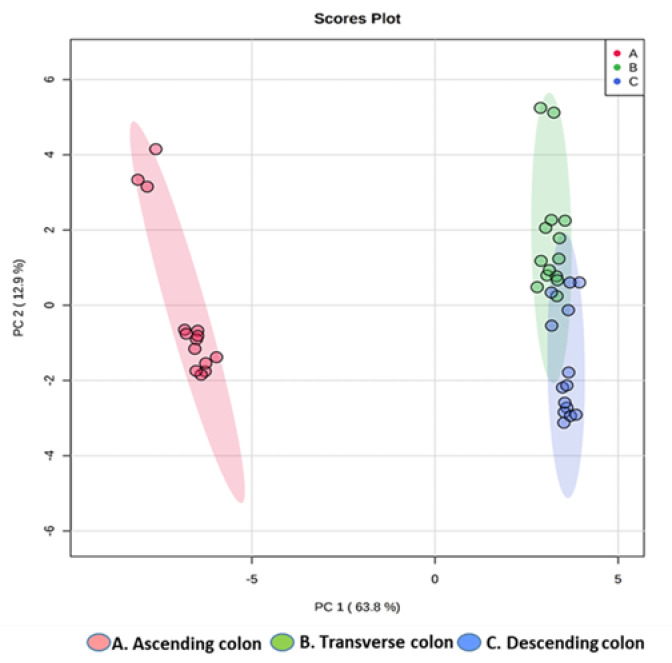
Principal component analysis (PCA) score plot of HPP-treated onion fermentation products. The parameters for these models were: R2 = 0.934, Q2 = 0.921. A Student’s t-test at 95% level (*p* < 0.05) was used to select the most important variables in each model.

Based on a total of 34 features found by the LC/MS negative ionization mode of the HPP-treated onion colonic fermentation samples, among which 11 were phenolic compounds precursors and 23 were phenolic metabolites (Table 2), a PCA model was built showing a clear separation of components into three different clusters, indicating a different composition in phenolic compounds and in their corresponding metabolites at each of the three regions of the colon (Figure 4). Data variability was mainly given by the first component (PC1) (63.8%). The multivariate model showed good quality in terms of variance explained (R_2_ = 0.934) and variance predicted (Q_2_ = 0.921).

### 2.3. Modulation of Microbiota Population

**Figure 5:**
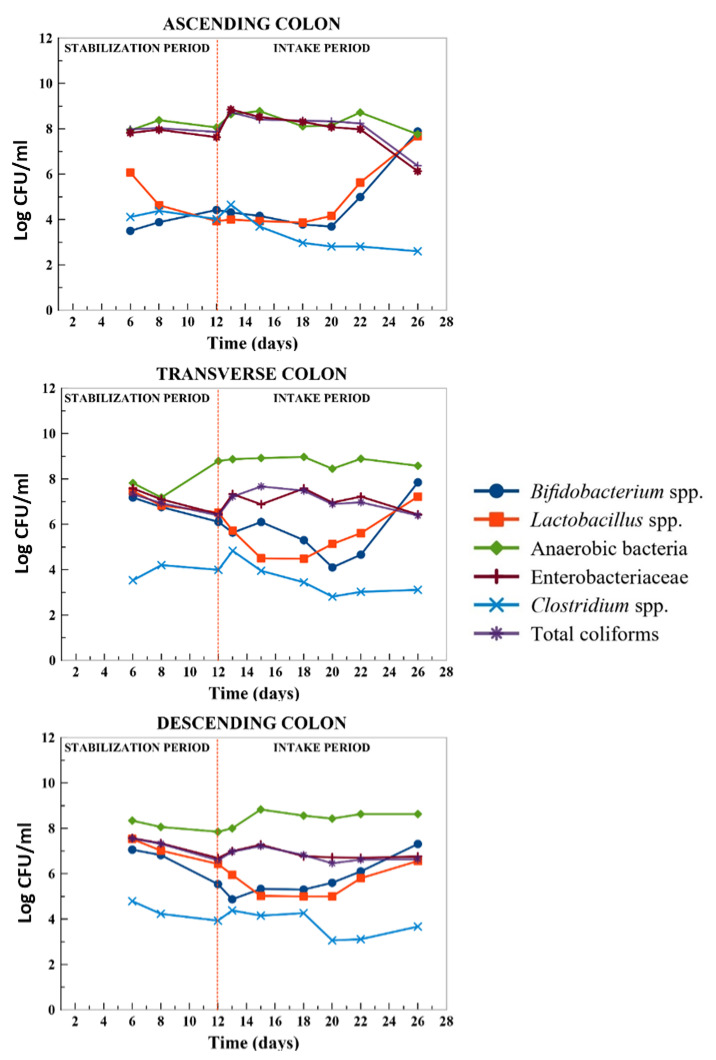
Changes in bacterial populations in the AC (ascending colon), TC (transverse colon), and DC (descending colon) during the stabilization period and intake period with HPP-treated onion: days 0 (13), day 2 (15), day 5 (18), day 7 (20), day 9 (22), and day 13 (26). Results are expressed as log CFU/mL (*n* = 2).

A bidirectional relation between phenolic compounds and microbiota has been suggested based on the role of colon microbiota in the metabolism of phenolic compounds and at the same time the influence of phenolic metabolites in the microbiota composition [65]. Thus, the influence of HPP-treated onion on gut microbiota population was evaluated by using microbiological counts methods, although more accurate molecular methods such as quantitative PCR and fluorescence in situ hybridization (FISH) with ribosomal RNA-targeted oligonucleotide probes exist. The colon microbial population analyzed was *Enterobacteriaceae* and anaerobic bacteria as indicators of total colon microbiota, *Bifidobacterium* spp. and *Lactobacillus* spp. considered beneficial bacteria, and *Clostridium* spp. and total coliforms as potential harmful colon bacteria (Figure 5).

The anaerobic bacteria content was stable in the three colon regions (AC, TC, DC) during the 14 days of feeding the gastrointestinal-colon fermentation system with the HPP-treated onion. A decrease of *Enterobacteriaceae* of 1.5 log CFU/mL in the AC compared to the baseline concentration was observed after the 14 days of the chronic feeding while remaining stable in the TC and DC (Figure 5). In addition, the level of *Clostridium* spp. decreased in the three colon regions, being more remarkable in the AC (~1.5 log CFU/mL). Furthermore, a decrease of total coliforms in the AC (1.5 CFU/mL) was observed meanwhile remained stable in the TC and DC (Figure 5).

With respect to the beneficial bacteria population, *Bifidobacterium* spp. and *Lactobacillus* spp. increased in the three colon regions and the major increment was observed in the AC after the first 4 days of feeding the system with HPP-treated onion (3 and 3.5 log CFU/mL, respectively) (Figure 5).

Therefore, HPP-treated onion seemed to favor the microbiota composition balance through the increase in *Bifidobacterium* spp. and *Lactobacillus* spp. and a tendency to decrease the level of *Enterobacteria*, total coliforms, and *Clostridium* spp. Different studies have suggested that phenolic compounds supplied through different dietary sources and their corresponding colonic bacterial metabolites may exert selective prebiotic effects and antimicrobial activities against gut pathogenic bacteria [13,65].

In a study by Duque et al. [66], treatment with fresh and pasteurized orange juice allowed the increase of 1 log CFU of *Bifidobacterium* spp. and *Lactobacillus* spp. in the three colon regions using the SHIME, an in vitro dynamic fermentation model. In addition, *Bifidobacterium* spp. increased significantly (*p* < 0.05) after the batch-culture fermentation of fresh ‘Renetta Canada’ and ‘Golden Delicious’ apples compared to cellulose and to a greater extent than the increase observed after inulin (a known prebiotic) administration [23]. The reduction of *Enterobacteria* and *Clostridium* spp. populations by several phenolic compound food sources has been reported. A significant reduction in the *Enterobacteriaceae* population was observed in the ascending and descending colon after the in vitro fermentation of cranberry and grape seed extract [38]. On the other hand, an intervention study of flavan-3-ols in cocoa (catechin and epicatechin) in healthy volunteers limited the growth of the *Clostridium histolyticum* group [31]. Moreover, a red wine extract subjected to an in vitro fermentation process showed a slight inhibition of the *Clostridium histolyticum* group [67].

The increase of *Bifidobacterium* spp. and *Lactobacillus* spp. after chronic feeding with HPP-treated onion observed in the present study could be related with the accumulation of the major metabolites in the fermentation products derived from quercetin glycosides, such as 3,4-dihydroxyphenylacetic rapidly transformed to 3-hydroxyphenylacetic acid, and other monohydroxy-phenylacetic acids. Other authors have found significant increases of 61% and 35% in the proliferation of *Bifidobacterium longum* after the fermentation of quercetin and 3-hydroxyphenylacetic acid, respectively, by the gut microbiota [24]. It is important to highlight the high concentration of non-digestible dietary fiber of the HPP-treated onion (19.11 ± 0.50 mg/100 g dw) (Table 1) that reaches the colon and could be another important factor that modulates the gut microbial growth.

### 2.4. Short-Chain Fatty Acids (SCFAs) Content

**Figure 6:**
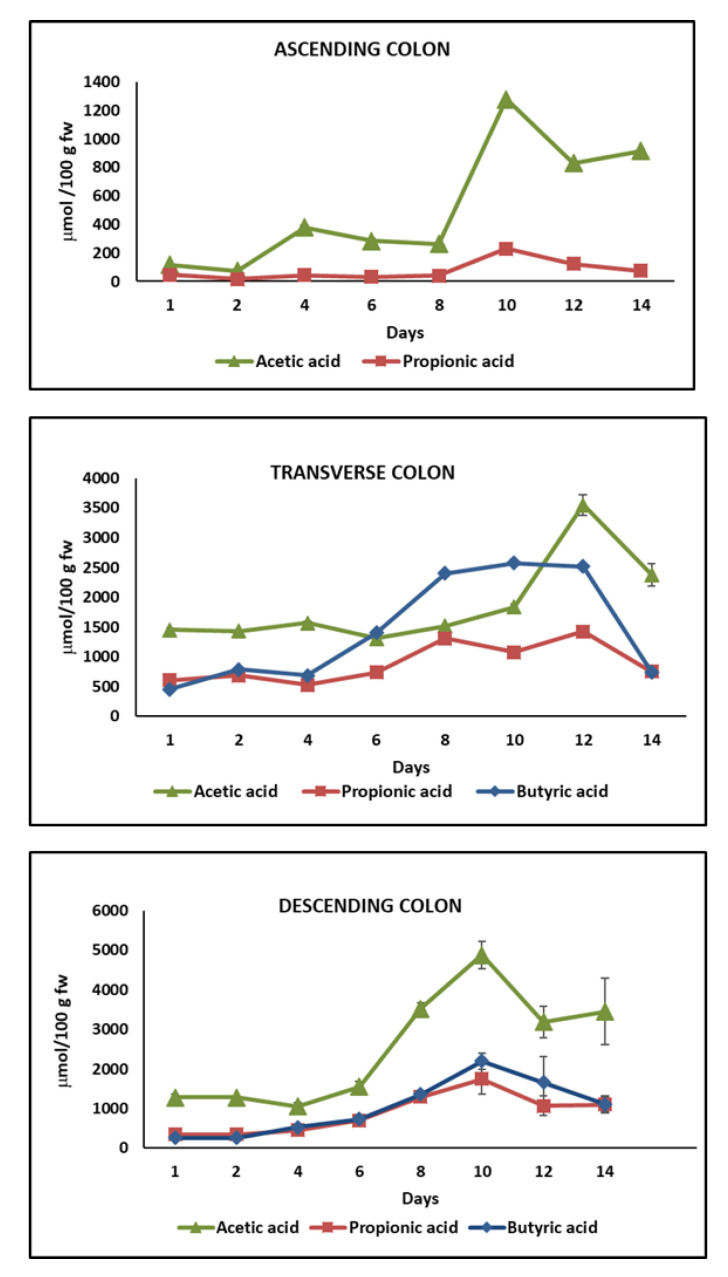
Concentration (µmol/100 g fw of slurry) of the main short-chain fatty acids (SCFAs) in the ascending colon (AC), transverse colon (TC), and descending colon (DC) during the colonic fermentation of HPP-treated onion. Data are the mean values ± standard deviation of three different determinations (*n* = 3).

The impact of chronic feeding with HPP-treated onion on colon microbiota activity using the DGID-CF simulator can be also evaluated by observing the formation of SCFAs. The main SCFAs found were acetic acid (C2:0), propionic acid (C3:0), and butyric acid (C4:0) (Figure 6), and they represented more than 95% of total fatty acids analyzed. Other minor fatty acids determined were isobutyric acid (iso C4), valeric acid (C5:0), caproic acid (C6:0), and heptanoic acid (C7:0). The individual and total SCFAs content was higher in the TC and DC than in the AC. The total concentration increased progressively in the three colon regions (AC, DC, TC) during the intake period reaching a peak of total concentration at day 10 in the AC (1506.87±17.78 µmol/100 g fw) and DC (10143.71±1150 µmol/100 g fw), while in the TC, total peak concentration was achieved at day 12 (5695.18 ± 131.13 µmol/100 g fw) (Figure 6).

The lowest concentration of total SCFA was found in the ascending colon (AC) during the 14 days of the study (Figure 6) with acetic acid being the major SCFA followed by propionic acid. Both acetic and propionic acid content increased progressively during the chronic feeding with HPP-treated onion from day 0 (117.38 ± 9.02 and 45.43 ± 6.14 µmol/100 g fw, respectively) to day 10 (1279 ± 0.21 and 228.59 ± 17.55 µmol/100 g fw, respectively) in the AC (Figure 6).

In the TC and DC, in addition to acetic and propionic acid, butyric acid was also detected as one of the main SCFAs in the TC from day 6 to day 10 of the assay and its concentration was approximately 40% and 58% higher than acetic and propionic acids, respectively (Figure 6). In the DC, the major compound during the 14 days of intake period was acetic acid, while not significant differences were found between butyric and propionic acid content (Figure 6).

The concentration of the three SCFAs increased progressively during the intake period, reaching peak concentration at day 10 where the content of acetic acid (4853.89 ± 347 µmol/100 g fw), butyric acid (2197.93 ± 382 µmol/100 g fw), and propionic acid (1747.19 ± 202 µmol/100 g fw) was 4, 5, and 8 times higher, respectively, than their concentration at day 0 (Figure 6).

**Figure 7:**
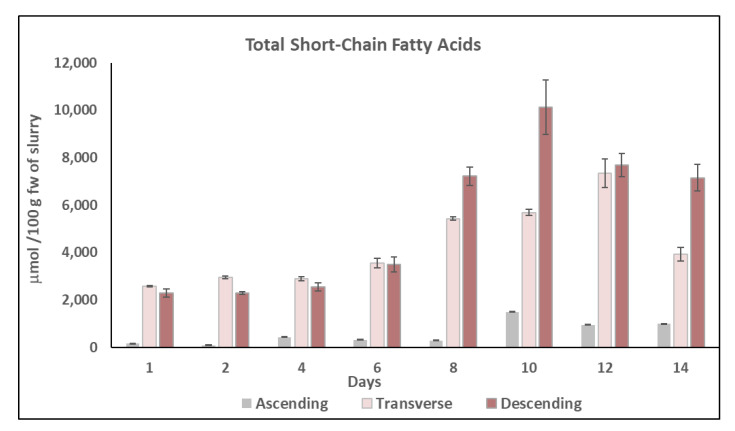
Total concentration (µmol/100 g fw of slurry) of short-chain fatty acids (SCFAs) in the ascending colon (AC), transverse colon (TC), and descending colon (DC) during the colonic fermentation of HPP-treated onion. Data are the mean values ± standard deviation of three different determinations (*n* = 3).

Therefore, chronic feeding with HPP-treated onion resulted in a significant increase of total SCFAs with a peak level at day 10 in the three colon regions: AC (1506.88 ± 17.8 µmol/100 g fw of slurry), TC (5695.18 ± 131 µmol/100 g fw of slurry), and DC (10,143.71 ± 1150 µmol/100 g fw of slurry) (Figure 7), with acetic and propionic acid being the major SCFAs in AC and DC, while butyric acid was the major SCFA in TC (Figure 6).

Acetic, propionic, and butyric acids were found to be the main SCFAs produced during the fermentation process using an in vitro model of samples rich in phenolic compounds such as cranberry and grape seed extracts by the gut microbiota. In addition, in this type of assay, the content of SCFAs was higher in the DC than in the AC [38]. Considering in vivo assays, the group of rats fed with an onion supplemented diet produced significant higher concentrations of SCFAs than the control group fed with a traditional rat diet without onion [68]. Furthermore, in vitro gut fermentation studies carried out with quercetin, the main flavonol found in onions, resulted in a high accumulation of propionic acid; meanwhile, the fermentation of caffeic acid showed the increase of butyric acid respective to other SCFAs [24]. However, in the present study, the main SCFAs were acetic acid followed by propionic acid in AC and DC except in the TC where butyric acid was the main compound from day 6 to day 10 of the study.

The increase of SCFA concentration during chronic feeding with HPP-treated onion in a DGID-CF simulator indicates that this intake could improve the host health because SCFAs play an important role in the production of gut hormones, act in the brain to regulate food intake, exert protective effects against enteric pathogen colonization and infection, and play an important role in the prevention and treatment of metabolic diseases and the prevention of cardiovascular disease, among other functions [26]. Moreover, the increasing concentration of butyric acid in the TC due to the consumption of HPP-treated onion indicates an important protection against colon cancer [28].

As a consequence of the degradation of proteins and amino acids by gut microbiota, small amounts of fatty acids such as isoC4, C5, C6, and C7 were found mainly in the DC. In both the TC and the DC, a peak level was observed at day 10 of the study, C5 being the main compound in the TC (181.07 ± 3.97 µmol/100 g fw of slurry), while in the DC, it was C6 (1014.06 ± 150 µmol/100 g fw of slurry). Contrary to the present study results, other authors only observed an increment of isoC4 in the AC during an in vitro gut fermentation of cranberry and grape seed polyphenols [38]. In addition, non-significant differences were found in the C4, C5, isoC5, C6, and C7 content in the cecum of Wistar rats fed with blackcurrant, blackberry, and raspberry extracts [69].

## 3. Materials and Methods

### 3.1. Reagents

Methanol LC/MS grade was acquired from J.T. Baker (Deventer, The Netherlands). The rest of the solvents employed were HPLC-grade from Lab-Scan (Dublin, Ireland). Commercial standards of C2:0, C3:0, C4:0, and C5:0 fatty acids were acquired from Applied Science Laboratories Inc. (State College, PA, USA). The rest of the commercial standards and the chemical products employed in the different analyses carried out were supplied by Sigma Aldrich (St. Louis, MO, USA).

### 3.2. Raw Material

Onions (*Allium cepa* L. var. cepa, ‘Recas’) from Carabaña (Madrid, Spain) were purchased from a local supermarket located in Madrid and stored at 4 °C until processed.

External layers were removed, and bulbs were cut into 10 mm cubes, packaged (200 g) in low permeability plastic bags (BB4L, Cryovac, Barcelona, Spain), and sealed with light vacuum.

### 3.3. High-Pressure Processing (HPP)

The onion bags were treated at 400 MPa for 5 min at 25 °C. These treatment conditions were selected due to the good results in terms of health-promoting characteristics demonstrated for this HPP-treated onion by in vitro and in vivo assays carried out with Wistar rats [40,41,70,71]. The hydrostatic pressure unit was formed by a vessel of 1925 mL capacity and it was able to work to a maximum pressure of 800 MPa and a maximum temperature of 70 °C (High Pressure Iso-Lab System, Model FPG7100:9/2C, Stansted Fluid Power LTD., Essex, UK). Two bags of packaged onions were placed into the vessel unit filled with water as a pressure-transmitting medium. The compression and decompression rates were both 3 MPa/s. Because of adiabatic compression, the maximum temperature in the vessel was 25 °C at 400 MPa. Pressure, time, and temperature were controlled by a computer program, being constantly monitored and recorded during the process.

Onion treated by high-pressure processing (HPP-treated onion) was immediately frozen with liquid nitrogen and stored at −80 °C until lyophilization (100 mTorr, −90 °C) using a lyophilizator model Lyoalfa (Telstar, Barcelona, Spain). Lyophilized samples were pulverized in an ultracentrifugal grinder ZM 200 (Retsch GmbH, Haan, Germany) to obtain a fine powder (particle size ≤ 0.5 mm), and maintained at −20 °C until analyzed.

### 3.4. Characterization of HPP-Treated Onion Powder

The effect of HPP at 400 MPa/25 °C/5 min on physicochemical and chemical characteristics of the ‘Recas’ onion has been previously studied [46,70,71]. The physicochemical and chemical characteristics of HPP-treated onion powder are shown in Table 1. Titratable acidity, pH, total vitamin C, and ascorbic acid were determined according to [45]. Sugars and organic acids content were analyzed according to Colina-Coca et al. [71]. Soluble proteins were analyzed according to the BioRad assay based on Bradford [72]. Soluble, insoluble, and total fiber (AOAC 991.43, 2012) were also determined.

### 3.5. Dynamic In Vitro Gastrointestinal and Colonic Fermentation (DGID-CF) Model

**Figure 8:**
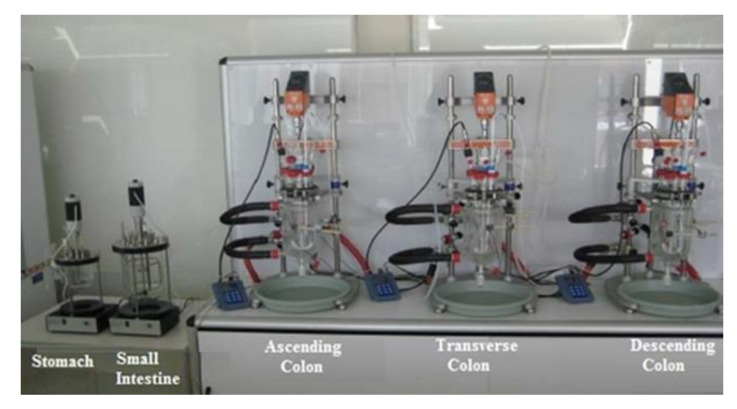
Dynamic in vitro model system to simulate the whole digestion process, gastrointestinal digestion, and colonic fermentation (DGID-CF) developed by AINIA (Valencia, Spain).

A dynamic in vitro model system able to simulate the complete digestion process, i.e., gastrointestinal digestion and colonic fermentation (DGID-CF) was developed by AINIA Technologic Center (Valencia, Spain) based on different authors [39,49,73,74]. This system consists of five vessels to simulate the physiological conditions taking place during digestion in the stomach, small intestine, and ascending (AC), transverse (TC) and descending colon (DC). All the vessels were protected from light and connected by peristaltic pumps working semi-continuously in the stomach and small intestine, and continuously in the three colon regions (Figure 8). The five vessels were under agitation (150 rpm) and the whole process conditions, pH values, residence times, temperature (37 °C) and volume capacities, were computer-controlled (Figure 8). Anaerobiosis in the five vessels was maintained by flushing gaseous N_2_ for 15 min twice a day.

The gastrointestinal digestion (stomach and small intestine) conditions were similar to Minekus et al. (2014) [75]. This system was previously employed to study the bioaccessibility of flavonols in HPP-treated onion by Fernández-Jalao et al. (2017) [46]. Briefly, the gastric digestion step was simulated by the addition of 60 mL of a 0.03% (*w*/*v*) pepsin solution (2100 units/mg) made with simulated gastric electrolyte solution to the stomach vessel. In contrast, the small intestine digestion was simulated by the addition of 240 mL of a solution containing pancreatin (0.9 g/L) and fresh bile (6 g/L) made with a simulated intestinal electrolyte solution in distilled water. The pH in the stomach (pH 2) and small intestine (pH 5–6) was adjusted with hydrochloric acid 1M or sodium bicarbonate, respectively. Once the gastric digestion (2 h) and small intestine digestion was finished (6 h), the intestinal digestion was incorporated three times a day to the ascending colon reactor.

A pool of feces from five healthy volunteers with a varied diet, no history of gastrointestinal disorders, no smokers, no food allergies, not taking any vitamins or supplements, and having taken no antibiotics in the three months prior to start the study was used. Samples were collected on site on the day of the experiment and were used immediately. All volunteers gave their informed consent for the use of their feces in the study. The study was conducted in accordance with the Declaration of Helsinki, and the protocol was approved by the Ethics Committee of the AINIA Technology Centre (AINIA procedure no. 1180/15).

Prior to the inoculation with 20 mL of human fecal slurry (20%, *w*/*v*) obtained by mixing the fresh feces with sodium phosphate (pH 7), the colon vessels were filled and pre-conditioned with a nutritive medium in a volume of 1000 mL in the AC, 1600 mL in the TC, and 1200 mL in the DC [76,77]. The composition for 1 L of the nutritive medium was 1 g of arabinogalactan, 0.2 g of pectin from apple, 1 g of xylan, 3 g of potato starch, 0.4 of glucose, 3 g of yeast extract, 1 g of peptone, 4 g of mucin, 0.5 g of L-cysteine, 1.5 g of NaHCO_3_, 0.69 of MgSO_4_·H_2_O, 0.5 g of KH_2_PO_4_, 0.5 g of K_2_HPO_4_, 0.08 g CaCl_2_, 0.005 g of FeSO_4_·7H_2_O, 0.002 g of MnSO_4_·H_2_O, 0.0006 g of ZnSO_4_·7H_2_O, 0.0001 g of CoSO_4_·6H_2_O, 10 µL of vitamin K1, 1 mL of Tween 80 and 4 mL of resazurin solution (0.025%, *w*/*v*) as an anaerobic indicator, and distilled water. The medium was sterilized at 121 °C for 15 min and oxygen was eliminated by flushing nitrogen before being placed in the three colon reactors. The pH of the medium of each reactor was adjusted and controlled at 5.5–6 for AC, 6–6.4 for TC and 6.4–6.8 for DC and maintained under anaerobic conditions by continuously flushing N_2_.

**Figure 9:**
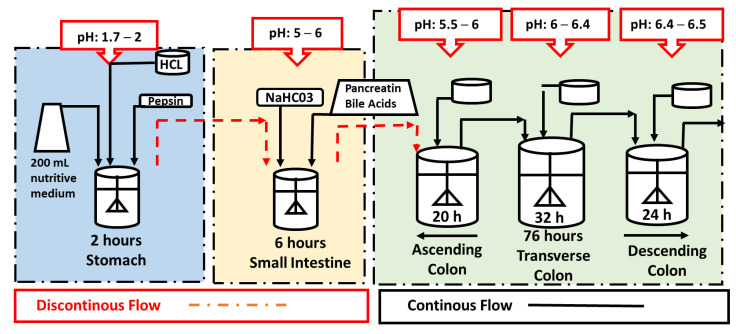
Experimental conditions of the in vitro gastrointestinal digestion and colonic fermentation in a dynamic model digestor (AINIA, Valencia, Spain).

The experimental procedure consisted of two phases. An initial stabilization period (basal) of 12 days allowed the intestinal microbiota to adapt to the nutritional and physicochemical conditions present in the three colon vessels (AC, TC, and DC). During this period, 200 mL of nutritive medium was added three times a day to the stomach reactor to feed the system (Figure 9). The second phase corresponded to the intake period, which consisted of adding 27 g of HPP-treated onion to the stomach vessel once a day for 14 days, together with 200 mL of nutritive medium simulating a chronic intake. In addition, the system was fed twice a day only with 200 mL of nutritive medium. The volume and transit time of each compartment of the DGID-CF simulating the in vivo residence time were: 260 mL and 2 h in the stomach, 460 mL and 3 h in the small intestine, 1000 mL and 20 h in the AC, 1600 mL and 32 h in the TC, and 1200 mL and 24 h in the DC (Figure 9).

During the experimental set up, samples of 50 g from the three colon reactors (AC, TC, and DC) were collected once a day during the 14 days of experiment to study the metabolic activity of intestinal microbiota (phenolic metabolites and short-chain fatty acid analysis). All these samples were quicky aliquoted and stored at −20 °C in darkness until they were transported from AINIA to ICTAN under controlled freezing conditions. On arrival, they were immediately stored at −80 °C for one week and then subjected to lyophilization (Lyophilizator model Lyoalfa, Telstar S.A, Barcelona, Spain). The lyophilized samples were homogenized manually with a domestic grinder and stored at −20 °C until analysis. For the evaluation of colonic microbiota modulation, samples of 5 g from the three colon reactors (AC, TC, and DC) were collected in aseptic conditions at regular time points during the stabilization (0, 6, 8, 10, and 12 days) and intake period (1, 3, 5, 7, 9, and 13 days), immediately stored at 4 °C, and analyzed within two hours at the AINIA microbiology laboratory. Two different gastrointestinal digestion-colon fermentation digestions were carried out.

### 3.6. Analysis of Phenolic Compounds and Metabolites by HPLC-DAD-ESI-QTOF-MS/MS

#### 3.6.1. Phenolic Compounds in the HPP-Treated Onion Powder

The extraction of phenolic compounds of the undigested HPP-treated onion powder was carried out according to a procedure previously described [46]. Briefly, 0.5 g of HPP-treated onion was mixed with 12.5 mL of extraction solvent (methanol/water, 80:20, *v*/*v*) and homogenizer using an ultrahomogenizer at 8000 rpm for 4.5 min (model ES-270, Omni International Inc., Gainesville, VA, USA). The mixtures were centrifuged (8000× *g*, 4 °C, 15 min) using a refrigerated centrifuge (Thermo Scientific Sorvall, mod. Evolution RC, Thermo Fischer Scientific Inc., Waltham, MA, USA). The pellet was re-extracted with 12.5 mL of extraction solvent and centrifuged again. Finally, the two supernatants were combined, evaporated at 40 °C using a vacuum evaporator, reconstituted with 10 mL of methanol, and stored at −20 °C until analysis by HPLC-DAD- ESI-QTOF-MS/MS. Analysis was performed in triplicate (*n* = 3).

#### 3.6.2. Phenolic Compounds and Metabolites in the Colon Fermentation Products

For the extraction of phenolic compounds and metabolites from the colonic fermentation products (ascending, transverse, and descending colon), 25 mg of lyophilized samples was mixed with 1.5 mL of methanol/acidified water (0.1% formic acid) (80:20 *v*/*v*). The mixtures were vortexed for 1 min, sonicated for 5 min, and centrifuged at 11,400× *g* (Thermo Scientific Sorvall, mod. Evolution RC, Thermo Fisher Scientific Inc., Waltham, MA, USA) for 15 min at 4 °C. The supernatants were separated and centrifuged twice more in the same conditions. The final supernatant (1 mL) was diluted with 2 mL of ultrapure water (or 2 mL of methanol/water, 80:20 (*v*/*v*) for detection and quantification of quercetin aglycone). The extraction was done in triplicate. One quality control (QC) (mix of extracts of all samples) and one pool sample by zone (mix of extracts of all samples from each colon region: ascending, transverse, and descending) were also prepared. The extracts were filtered through a 0.22 µm filter before their analysis by HPLC-DAD-ESI-QTOF-MS/MS. Analysis was performed in triplicate (*n* = 3).

#### 3.6.3. HPLC-DAD-ESI-QTOF-MS/MS Analysis of Phenolic Compounds and Metabolites. Metabolomic Analysis by a Targeted Approach

The separation, identification, and quantitation of phenolic compounds and their metabolites was achieved by high performance liquid chromatography-mass spectrometry system with a diode array detector (DAD) and coupled to an Agilent 6530 Accurate-Mass Quadrupole Time-of-Flight (Q-TOF) MS system equipped with an Agilent Jet Stream dual electrospray ionization (ESI) source (HPLC-DAD-ESI-QTOF-MS/MS)(Agilent Technologies Inc., Santa Clara, CA, USA). The HPLC system was equipped with a quaternary pump (G1311A) with an integrated degasser (G1322A), thermostated automatic injector (G1367B), thermostated column module (G1316A), and a diode detector array (DAD) (G1315B) (Agilent Technologies, Waldbronn, Germany). Separation was carried out on a 2.0 × 50 mm id., 3.0 μm particle size, Luna C18 column (Phenomenex, Torrance, CA, USA). The mobile phase consisted of a linear gradient of 0.1% formic acid in Milli-Q-water (A) and 0.1% formic acid in acetonitrile (B) as follows: 0 min, 95% A; 15 min, 80% A; 25 min, 50% A; 30 min, 20% A; 40 min, 95% A. The flow rate was fixed at 0.4 mL/min and the injection volume was 10 µL. Runs were monitored at 360 nm, 320 nm, and 280 nm.

To identify the phenolic compounds precursors and their possible metabolites, total ion spectra were collected in the range *m*/*z* 100–1000 in negative mode. Nitrogen was used as a drying, collision, and nebulizing gas. The drying gas temperature and flow rate were 225 °C and 10 L/min, respectively. The sheath gas temperature and flow rate were 300 °C and 10 L/min. The nebulizer gas pressure, skimmer voltage, octopole RF, and fragmentor voltage were 45 psi, 65 V, 750 V, and 125 V, respectively. The capillary voltage was set at 3 kV. The MS/MS collision energy was set at 15 eV.

Data were acquired and analyzed using a Masshunter Qualitative Analysis B.07.00 software and Masshunter Profinder B.08.00 software (Agilent Technologies Inc., Santa Clara, CA, USA), respectively. The identification was possible by comparison with the mass spectral data generated by external standards, the data from databases (MassBank, Pubchem, Phenol explorer, MoNa database), and a personal accurate mass database built using the information about mass data of the main phenolic compounds present in onion and their possible metabolites obtained from the literature. The raw data collected by LC-MS was cleaned of background noise and unrelated ions by the molecular feature extraction (MFE) algorithm using the Mass Hunter Qualitative Analysis Software B.06.00 (Agilent Technologies, Inc, Santa Clara, CA, USA). The MFE then created a list of the ion characteristics for the phenolic compounds and their corresponding metabolite profiles; each compound was described by mass, retention time, and abundance. Features post-acquisition processing was performed in Agilent Profinder B.08.00 (Agilent Technologies Inc., Santa Clara, CA, USA). Mass and retention time alignment followed by a filter-by-frequency post-processing was done to retain only those features that were present in >70% of samples within at least one treatment. Relative standard deviation (%RSD) of the concentration of the compounds in the QCs (quality control) samples was calculated, and only compounds below the limit (30%) were considered for the data treatment.

Quantification of phenolic compounds precursors and metabolites was performed in the QTOF in MS1 mode using external calibration curves that were each systematically injected 10 runs of the injection sequence. Standard calibration curves of five points were made for each compound commercially available in the range of 5 to 0.005 µg/mL. When no commercial standard was available, the compounds or the metabolites were quantified using structurally related commercial standards. The quality control samples were prepared mixing 10 µL of each sample extract. Analysis was performed in triplicate (*n* = 3).

### 3.7. Microbial Analysis

Although there are accurate molecular methods such as quantitative PCR and fluorescence in situ hybridization (FISH) with ribosomal RNA-targeted oligonucleotide probes, in the present study bacterial count methods were employed. Thus, samples (5 g fw of slurry) from the three colon vessels (AC, TC, and DC) during the stabilization (at 0, 6, 8, and 12 days) and intake period (at 0, 2, 5, 7, 9, and 13 days) were aseptically transferred to tubes containing Anaerobe Basal Broth (Oxoid, Thermo Scientific, Basingstoke, UK) transported immediately to the microbiology laboratory of AINIA (Valencia, Spain), and analyzed within two hours [78]. The isolation and enumeration of specific groups of bacteria from the fermentation products were done using selective and differential growth media and suitable incubation conditions [79]: VRBD Agar (Merck-Millipore, Burlington, MA, USA) for Enterobacteriaceae; VRBL Agar (Merck-Millipore) for total coliforms; TSC Agar (Oxoid) for *Clostridium* spp.; Tos-propionate Agar (Merck-Millipore) for *Bififobacterium* spp.; Schaedler Anaerobe Agar (Oxoid) for total anaerobes; and MRS Agar (Oxoid) for *Lactobacillus* spp. All plates were incubated at 37 °C in anaerobic conditions for 24 h (Enterobacteriaceae, *Clostridium* spp., and total coliforms), 48 h (*Bififobacterium* spp. and *Lactobacillus* spp.), and 72 h (total anaerobes). The identification of Lactobacillus microorganisms from MRS-agar plates were carried out by MALDI-TOF mass spectrometry [80]. Anaerobic bacteria and Enterobacteriaceae were analyzed as indicators of total colonic microbiota, total coliforms, and *Clostridium* spp. as a part of regular microbiota composition. Although they are associated with potential harmful effects, *Bifidobacterium* spp. and *Lactobacillus* spp. were associated with the beneficial microbiota of the colon. Analysis was performed in triplicate (*n* = 3).

### 3.8. Analysis of Short-Chain Fatty Acids (SCFAs)

The SCFAs were derivatized using the methodology previously described by Castro-Gomez et al. [81]. Briefly, 100 mg of lyophilized colonic fermentation products dissolved in 400 L of Milli-Q water was mixed with 2.50 mL of sodium methoxide 0.5 M solution in methanol and 50 µL of nonanoic acid (C9:0) and tritridecanoine (C13:0-TAG) solution as internal standards, 1 mg/mL. The samples were heated at 80 °C, 10 min, and 1300 rpm using an incubator with shaker function (TR100-G; JP Selecta, Barcelona, Spain). The samples were cooled in ice for 5 min, and then 1.83 mL of dimethylsulfoxide (DMSO) and 3 mL of H_2_SO_4_ (1M) were added. The samples were incubated again (60 °C, 30 min, and 1300 rpm) and cooled later. Then, 1 mL of hexane and 7.5 mL of 6% Na_2_CO_3_ solution were incorporated into samples and the mixtures were centrifuged at 0 °C, 3500 rpm, and 10 min, using a refrigerated centrifuge (Hettich Universal centrifuge 320R, Tuttlingen, Germany). The upper layer of hexane was transferred to a vial for GC-MS analysis. The same derivatization method was applied to the reference mix (1 mg/mL) prepared from commercial standards of fatty acids (C4:0, C5:0, C7:0, C8:0, and C9:0).

The analysis of fatty acid methyl esters (FAMEs) obtained was performed in an Agilent 6890 Series gas chromatograph (Agilent Technologies, Heilbronn, Germany) coupled to an Agilent 5973 Series mass spectrometer (Agilent Technologies, Heilbronn, Germany). Chromatographic separation was performed in a CPSil-88 capillary column (100 m × 0.25 mm × 0.2 µm) (Chrompack, Middelburg, The Netherlands). Helium (purity 99.999%) was used as a carrier gas with a column inlet pressure of 30 psi. The injection volume was 1 µL and the split ratio was 1:25. The temperature program was as follows: 1 min at 70 °C, first ramp 7 °C/min to 170 °C, and secondary ramp at 10 °C/min to 230 °C. The total analysis run was 76.29 min. MS detector conditions were as follows: transfer line temperature 250 °C, source temperature 230 °C, and quad temperature 150 °C. The mass spectrometer was operated under electron impact ionization at 70 eV; it was used in total ion current (TIC) mode and scanned the mass range from 40 to 500 *m*/*z*. For SCFA identification of colonic fermentation samples, the retention time and mass spectra were compared with those obtained in a mix of commercial standards and data from the National Institute of Standards and Technology library (NIST, 2.1.0 version; Gaithersburg, MD, USA). Quantitation was achieved using response factors calculated from the internal standards. Analysis was performed in triplicate (*n* = 3).

### 3.9. Statistical Analysis

The results shown represent mean values ± standard deviation (SD). These results were calculated from the mean value obtained in at least three separate experiments (*n* = 3). To study statistically significant differences (*p* ≤ 0.05) in flavonols and their metabolite content, short-chain fatty acids production, and microbiota composition in the three colon regions at different fermentation days, one-way analysis of variance (ANOVA) was applied followed by Tukey’s b post hoc test. Levenne’s test was applied to verify the homogeneity of the variances. All analyses were performed using the IBM SPSS Statistics 23 Core System (SPSS Inc., an IBM Company, Armonk, NY, USA).

Multivariate analysis based on principal component analysis (PCA) was applied to assess the quality of data obtained by HPLC-DAD-ESI-QTOF-MS/MS using the MetaboAnalyst 4.0 software (MetaboAnalystR package) (XiaLab, MacGill University, Montreal, QC, Canada) [82]. Pareto was used as a scaling method and a logarithmic transformation was performed in order to approximate a normal distribution. In order to evaluate the quality of analytical data and to remove possible outliers, a PCA was performed from fecal samples and quality controls (QCs). The robustness of analytical procedure was proven by the clustering of the QC samples (data not shown), which reflects the system’s stability and performance and the reproducibility of the sample treatment procedures.

## 4. Conclusions

To our knowledge, there are no published studies about the effect of a natural food source of flavonols, such as an onion product treated by high-pressure processing (HPP-treated onion) and subsequently lyophilized, on human gut microbiota metabolism by the simulation of chronic feeding with 27 g/day for 14 days using a dynamic in vitro gastrointestinal digestion and colon fermentation (DGID-CF) simulator. This simulator was composed of different reactors that mimic the conditions of the stomach, small intestine, and the three colon regions, ascending (AC), transverse (TC), and descending colon (DC).

Chronic feeding with 27 g/day of HPP-treated onion produced a significant accumulation of flavonols, mainly Q-3,4’-diglucoside, Q-4’-glucoside, Iso-4’-glucoside, and quercetin aglycone in the AC, that were metabolized by colonic microbiota, leading to a different flavonol metabolite profile depending on the colon region (AC, TC, DC). In the AC, the total metabolite content was 3.5 and 4 times lower than in the TC and DC, respectively. During the intake period, total metabolite content progressively increased, mainly in TC and DC, reaching a peak level at day 12 that was 2.5 times higher than at the beginning of the intake period. The main flavonol metabolites found in TC and DC have been related with important health-promoting properties and in descending order of concentration were 3-hydroxyphenylacetic acid, 4-hydroxyphenylacetic acid, 2-hydroxyphenylacetic acid, and 3-(3-hydroxyphenyl)-propionic and 3-(4-hydroxyphenyl)-propionic acids. Furthermore, the beneficial effect of chronic feeding with the HPP-treated onion was the increased level of *Bifidobacterium* spp. and *Lactobacillus* spp, considered part of beneficial colon bacteria, and the decreased *Enterobacteria*, total coliforms, and *Clostridium* spp. concentration as potential harmful bacteria. The beneficial effects on the composition of the microbiota produced by the HPP-treated onion can also be reflected in the increased production of SCFAs, mainly acetic acid followed by propionic acid in the AC and DC and butyric acid in the TC.

Data provided in the present study showed that HPP-treated onion is a good dietary source of flavonols that, in contact with human gut microbiota, resulted in phenolic metabolites and short-chain fatty acids production as well as the modulation of certain bacterial groups, changes that have been associated with health beneficial effects. However, more studies have to be done to know if the untreated onion would produce the same results as the HPP-treated onion.

## Figures and Tables

**Table 1:** Characterization and phenolic compound content of the HPP-treated onion powder.

Parameters	HPP-Treated Onion
**Physicochemical characteristics**	
pH	5.50 ± 0.04
Acidity (g citric acid/100 g dw)	0.60 ± 0.08
**Chemical characteristics**	
Ascorbic acid (mg/100 g dw)	147.78 ± 3.80
Vitamin C (mg/100 g dw)	170.60 ± 2.57
Proteins (g/100 g dw)	3.41 ± 0.27
**Fibre (mg/100 g dw)**	
Soluble	4.57 ± 0.15
Insoluble	14.54 ± 0.05
Total	19.11 ± 0.50
**Sugars (g/100 g dw)**	
Sacarose	3.58 ± 0.21
Glucose	11.95 ± 0.75
Fructose	22.15 ± 1.25
**Organic acids**	
Maleic acid (g/100 g dw)	1.48 ± 0.18
Citric acid (mg/100 g dw)	239.52 ± 2.31
Fumaric acid (mg/100 g dw)	12.09 ± 0.92
Acetic acid (mg/100 g dw)	5.21 ± 1.32
Pyruvic acid (mg/100 g dw)	80.23 ± 2.58
**Flavonols (mg/100 g dw)**	
Quercetin-3,7,4′-triglucoside	26.74 ± 2.39
Quercetin-7,4′-diglucoside	13.31 ± 1.06
Quercetin-3,4′-diglucoside	5090.47 ± 104.64
Quercetin-3-rutinoside	2.02 ± 0.37
Isorhamnetin-3,4′-diglucoside	174.46 ± 32.23
Quercetin-3-glucoside	108.88 ± 15.56
Quercetin-4′-glucoside	1015.55 ± 156.38
Isorhamnetin-3-glucoside	2.04 ± 0.31
Isorhamnetin-4′-glucoside	322.77 ± 48.46
Quercetin	5.57 ± 0.57
Isorhamnetin	3.35 ± 0.3
**Phenolic acids (mg/100 g dw)**	
Protocatechuic acid	0.56 ± 0.08
Gallic acid	1.23 ± 0.25
Salicylic acid	1.87 ± 0.07

Data expressed as mean ± SD (*n* = 4); dw = dry weight; HPP = 400 MPa/25 °C/5 min.

**Table 2:** Flavonols and phenolic metabolites identification by HPLC-DAD-ESI-QTOF-MS/MS in the fermentation products of HPP-treated onion powder subjected to a dynamic in vitro gastrointestinal digestion and colonic fermentation simulator.

Compounds	Formula	Mass	RT (min)	*m*/*z* (M − H)	Mayor ESI *m*/*z* [M − H]^−^ and Fragments
**Benzoic acid derivatives**					
3,4-Dihydroxybenzoic acid	C_7_H_6_O_4_	154.0243	1.91	153.0193	153.0199; 133.0260; **109.0291**; 91.0226; 81.0286
3,4,5-Trihydroxybenzoic acid (gallic acid)	C_7_ H_6_ O_5_	170.0191	2.10	169.0151	169.0153; **125.0229**; 107.0226; **96.9588**; 97.0280; 89.027; 68.9951
2-Hydroxybenzoic acid (salicylic acid)	C_7_H_6_O_3_	138.0322	3.03	137.0244	**137.0266**; 129.0563; 119.0363; 112.9852; **93.0347**; 89.0252; 68.9969
4-Hydroxybenzoic acid	C_7_H_6_O_3_	138.0322	3. 29	137.0257	137.0284; 124.0280; 112.9876; 108.1550; 98.957; **93.0345**
3,5-Dihydroxybenzoic acid	C_7_H_6_O_4_	154.0267	5.25	153.0193	**153.0267**; 137.0336; 131.0709; **109.0295**; **91.0038**; 68.9951
3-Hydroxybenzoic acid	C_7_H_6_O_3_	138.0323	5.53	137.0255	137.0251; 123.0451; 121.0295; **93.0351**; 91.0046
4-Hydroxy-3,5-dimethoxybenzoic acid (syringic acid)	C_9_H_10_O_5_	198.0528	6.20	197.0455	**197.0458**; 187.0215; 161.029; **153.0405**; 151.0416; **137.0319**
Benzoic acid	C_7_H_6_O_2_	122.0364	12.53	121.0294	121.0646; 102.9488; 89.0262; 77.0457
**Phenylacetic acid derivatives**					
3,4-Dihydroxyphenylacetic acid	C_8_H_8_O_4_	168.0425	2.78	167.0350	167.0343; **123.0447**; 137.0271; 96.4594; 61.9887
3-Methoxy-4-hydroxyphenylacetic acid (homovanillic acid)	C_9_H_10_O_4_	182.0579	2.66	181.0506	181.0584; 167.0358; **137.0305**; 123.0461; **122.0350**
4-Hydroxyphenylacetic acid	C_8_H_8_O_3_	152.0474	4.61	151.0407	151.0534; 137,0281; 128.0355; **121.0295**; 112.9862; **107.0497**; 96.9503
3-Hydroxyphenylacetic acid	C_8_H_8_O_3_	152.0474	6.12	151.0407	151.0406; **107.0512**; 112.9861; 108.0542; 119.0363; 65.0368
Hydroxyphenylacetic acid	C_8_H_8_O_3_	152.0474	7.47	151.0407	**151.0422**; 137.0302; 119.036; **107.0513**; **89.0253**
Phenylacetic acid	C_8_H_8_O_2_	136.0527	12.99	135.0451	135.0452; 128.0356; **117.2107**; 105.0039; **91.0479**; 65.0357
**Phenylpropionic acid derivatives**					
3,4-Dihydroxyphenylacetic acid) (dihydrocaffeic acid)	C_9_H_10_O_4_	182.0579	4.80	181.0471	181.0517; **137.0257**; **121.0274**; 119.0363; 112.9832; 109.0270
3-(3-Hydroxypheny)-propionic acid	C_9_H_10_O_3_	166.0630	9.31	165.0557	165.0417; **147.3718**; 137.0349; **121.0909**; 107.4270
3-(4-Hydroxypheny)-propionic acid	C_9_H_10_O_3_	166.0630	11.79	165.0557	165.0462; **148.3569**; **121.0439**; 107.0339; **92.8287**
Phenylpropionic acid	C_9_H_10_O_2_	150.0665	18.15	149.0607	149.0689; 105.2957; 133.4147; 88.1326, 74.0109
**Simple phenols**					
Phloroglucinol	C_6_H_6_O_3_	126.0335	0.77	125.0249	125.0214; 108.3153; 121.3252; **83.0178**
Catechol	C_6_H_6_O_2_	110.0366	2.66	109.0298	**109.0308**; 108.0225; 96.9602; **91.0153**; 89.0244
**Hydroxycinnamic derivatives**					
Caffeic acid	C_9_H_8_O_4_	180.0446	6.20	179,0356	179.0367; 161.0325; **135.04595**; 134.0401, **109.0283**; **61.9901**
Isoferulic acid	C_10_H_10_O_4_	194.0579	16.72	193.0506	193.0515; 178.9886; 149.2177; 96.9549
**Others**					
Dihydroquercetin	C_15_H_12_O_7_	304.0557	14,22	303.0510	303.0528; 285.0332; 260.9191; **151.9627**; **125.0254**; 110.9089
**Flavonols**					
Quercetin-3,7,4′-triglucoside	C_33_H_40_O_22_	788.2041	13.10	787.1937	787.1943; 770.3506; 671.1474; **625.1403**; 432.0439; 204.0667; 179.0487
Quercetin-7,4′-diglucoside	C_27_H_30_O_17_	626.1483	13.47	625.1404	**625.1301**; 606.1779; **463.0867**; 301.0267
Quercetin-3,4′-diglucoside	C_27_H_30_O_17_	626.1483	13.68	625.1404	625.1348; **463.0867**; 301.0370; 271.0606; 150.9868
Isorhamnetin-3,4′-diglucoside	C_28_H_32_O_17_	640.1656	14.34	639.1556	639.1556; **477.0853**; 313.0640; 303.0485
Quercetin-3-rutinoside	C_27_H_30_O_16_	610.1545	15.34	609.1457	**609.1471**; 553.3327; **463.0928**; 301.1196
Quercetin-3-glucoside	C_21_H_20_O_12_	464.0963	15.66	463.0884	463.0888; **343.0571**; **301.0319**; 271.0205; 229.0334
Quercetin-4-glucoside	C_21_H_20_O_12_	464.0963	17.28	463.0881	463.0897; 445.8403; 301.0342; 283.0054
Isorhamnetin-3-glucoside	C_22_H_22_O_12_	478.1122	17.33	477.1037	**477.1031**; 315.0414; 271.0043; 299.0117; 243.0197
Isorhamnetin-4-glucoside	C_22_H_22_O_12_	478.1122	18.21	477.1037	**477.1051**; 463.0893; 315.0393; 299.0150; 157.3284
Quercetin	C_15_H_10_O_7_	302.0381	19.98	301.0343	301.0340; 273.0260; **245.0376**; **178.9937**; **151.0054**;121.0313; 107.0123
Isorhamnetin	C_16_H_12_O_7_	316.0583	21.97	315.0501	315.0508; 299.9147; 285.9066; 258.9187; 161.0463

Bold number indicate that it is the majority fragment.

**Table 3:** Daily quercetin (Q) supply and evolution during a dynamic in vitro colonic fermentation of HPP-treated onion powder.

		Daily Quercetin Supply (µg)
		134.64
**Colon Region**	**Days**	**Quercetin (µg/100 g fw of Slurry)**
AC ^1^	4 d	360.22 ± 18.82 ^b^
	9 d	118.51 ± 13.44 ^a^
	14 d	529.73 ± 68.79 ^c^
TC ^1^	4 d	5.24 ± 1.74 ^b^
	9 d	1.70 ± 0.28 ^a^
	14 d	10.10 ± 0.51 ^c^
DC ^1^	4 d	0.89 ± 0.21 ^a^
	9 d	1.54 ± 0.2 ^a^
	14 d	2.37 ± 1.03 ^a^

^1^ Colon fermentation products were collected in the AC (ascending colon), TC (transverse colon), and DC (descending colon) at day 4, 9, and 14 of the fermentation studies. Data (µg/100 g fw of slurry) are expressed as mean ± standard deviation. Different small letters indicate significant differences (*p* < 0.05) between different sample collection days for the HPP-treated onion and colon regions (AC, TC, and DC).

## Data Availability

Not applicable.
